# Sensitivity to expression levels underlies differential dominance of a putative null allele of the *Drosophila t*β*h* gene in behavioral phenotypes

**DOI:** 10.1371/journal.pbio.3001228

**Published:** 2021-05-10

**Authors:** Christine Damrau, Julien Colomb, Björn Brembs

**Affiliations:** 1 Neurobiologie, Institut für Biologie, Fachbereich Biologie-Chemie-Pharmazie, Freie Universität Berlin, Berlin, Germany; 2 Institut für Zoologie–Neurogenetik, Universität Regensburg, Regensburg, Germany; Stony Brook University, UNITED STATES

## Abstract

The biogenic amine octopamine (OA) and its precursor tyramine (TA) are involved in controlling a plethora of different physiological and behavioral processes. The *tyramine-*β-*hydroxylase* (*t*β*h*) gene encodes the enzyme catalyzing the last synthesis step from TA to OA. Here, we report differential dominance (from recessive to overdominant) of the putative null *t*β*h*^*nM18*^ allele in 2 behavioral measures in Buridan’s paradigm (walking speed and stripe deviation) and in proboscis extension (sugar sensitivity) in the fruit fly *Drosophila melanogaster*. The behavioral analysis of transgenic *t*β*h* expression experiments in mutant and wild-type flies as well as of OA and TA receptor mutants revealed a complex interaction of both aminergic systems. Our analysis suggests that the different neuronal networks responsible for the 3 phenotypes show differential sensitivity to *t*β*h* gene expression levels. The evidence suggests that this sensitivity is brought about by a TA/OA opponent system modulating the involved neuronal circuits. This conclusion has important implications for standard transgenic techniques commonly used in functional genetics.

## Abstract 2

Differential dominance occurs when genes associated with several phenotypes (pleiotropic genes) show different modes of inheritance (e.g., recessive, dominant or overdominant) depending on the phenotype. This study reveals that differential sensitivity to gene expression levels can mediate differential dominance, which can be a significant challenge for standard transgenic techniques commonly used to elucidate gene function.

## Introduction

Pleiotropy is a central feature in genetics with pervasive implications for evolution [1–5]. Pleiotropic genes play an important evolutionary role not only because they create functional and developmental relationships among traits, but also because they can become relevant for the maintenance of genetic variability in a population [6,7]. While common, pleiotropy is not a universal property of all genes [8]. Pleiotropy is also a prerequisite for differential dominance. Differential dominance occurs when dominance patterns for a single locus vary among traits, e.g., the same allele may behave recessively in one trait and dominantly in another [4,9]. In wild populations, differential dominance is often accompanied by overdominance effects, which are thought to underlie the high level of heterozygosity found in these populations [4,6,7,9–11]. While heterozygosity tends to decrease in laboratory populations [12–18], the differential dominance effects may persist in pleiotropic genes, including overdominance.

Differential dominance, found to be ubiquitous in quantitative and population genetics studies [4,9,19], may potentially wreak havoc in functional genetics, where a common strategy is to introduce transgenic alleles into homozygous null mutant individuals [20–26]. For instance, if a mutation acts dominantly, rather than recessively, then the transgenic alleles will not rescue the phenotype even if the gene in question is responsible for it. In the case of overdominance, the outcome of such experiments may depend on the mechanism by which overdominance is achieved and could potentially range from no rescue to overdominant rescue, making these results difficult or impossible to interpret. Intermediate inheritance may make rescue experiments difficult to pin down statistically as successful or unsuccessful.

In the simplest case, the 2 alleles in question are a wild-type and a null mutant allele. In this arrangement, any differential dominance effects must be due to differential sensitivity of the phenotypes to gene dosage or gene expression levels or both. Therefore, such a situation is a good study case for investigating both the practical consequences for functional genetics studies and the mechanisms underlying the differential dominance phenomenon.

Because of the promiscuous role of biogenic amines in many different behavioral and physiological processes, the genes coding for their synthesis enzymes are prime candidates for pleiotropy and, hence, differential dominance. The biogenic amine octopamine (OA) is structurally and functionally related to vertebrate noradrenaline [27–29]. OA is synthesized from another biogenic amine, tyramine (TA), by *tyramine-*β*-hydroxylase* (*t*β*h*) [30]. OA plays an important role in the initiation and maintenance of motor programs in insects in general [31–35]. In skeletal muscles, OA concomitantly affects not only muscle tension [36] and relaxation rate [37], but also muscle metabolism: As a neurohormone released into the hemolymph, it mobilizes lipids and stimulates glycolysis [38,39]. OA appears to be involved in almost every behavioral and physiological process [40,41]. In *Drosophila*, the X-linked *t*β*h*^*nM18*^ mutant has been an important tool to understand the role of TA and OA in many behaviors such as egg laying [42–44], aggression [45,46], flight [47–50], and starvation resistance [51].

Loss-of-function *t*β*h*^*nM18*^ male mutants, with a complete depletion of OA, display reduced aggression: Their fight initiation latency is increased, while lunging and holding frequencies are decreased [46]. Furthermore, an acute silencing of octopaminergic neurons through the use of temperature-sensitive UAS-*Shi*^*ts*^ phenocopies the *t*β*h*^*nM18*^ mutants, indicating that the reduced aggression does not result from developmental defects in the mutants [45,52–54]. Interestingly, it was possible to rescue the aggression deficiency seen in *t*β*h*^*nM18*^ mutant flies by expressing *t*β*h* in a small subset of octopaminergic neurons [45]. These results suggest that the standard genetic rescue approach can be successful, at least in this phenotype, even with a pleiotropic gene. Some of us have shown previously that another phenotype, sugar sensitivity after starvation, can be analogously rescued [51].

In the present work we studied *t*β*h*-associated differential dominance and conducted rescue experiments using behavioral phenotypes as disparate as sugar sensitivity [55,56] and walking behavior in Buridan’s paradigm [57,58]. In Buridan’s paradigm, we evaluated walking speed, a temporal parameter of movement control, as well as stripe fixation, a spatial measure of movement control. Fixation of visual cues is increased at higher contrast conditions [59,60]. Interestingly, the sensitivity of the motion-sensitive neurons in the fly optic lobes was shown to increase when the fly is walking [61–64] or flying [65]. The gain increase in flight was found to be OA dependent [66,67]. The 3 phenotypes we investigated (sugar responsiveness, walking speed, and stripe fixation) exhibit differential dominance, and we use various transgenic rescue techniques commonly used to elucidate gene function to probe the consequences of differential dominance in functional genetics studies as well as potential mechanisms mediating the differential dominance phenomenon. We complement these experiments with OA receptor manipulations in order to isolate OA-dependent from TA-dependent effects and to explore whether such gene-dosage-independent manipulations may be a superior functional genetic approach to mutant/rescue experiments.

## Materials and methods

### Fly strains

*t*β*h*^*nM18*^ (Monastirioti et al., 1996; FBal0061578), *oamb* ([68]; *OctαR*, *oamb*^*286*^ FBti0038368, *oamb*^*584*^ FBti0038361), *honoka* (Kutsukake et al., 2000; *Oct-TyrR*, FBal0104701), *hsp*-*t*β*h* (Schwaerzel et al., 2003; FBal0152162), *Oct*β*2R*^Δ*3*.*22*^ and *Oct*β*2R*^Δ*4*.*3*^ (Damrau et al., 2014; CG6989, FBgn0038063), and w+;;UAS-*t*β*h* (Monastirioti, 2003; FBti0038601) were obtained from Henrike Scholz, Cologne, Germany; Hiromu Tanimoto, Martinsried, Germany; Andreas Thum, Konstanz, Germany; Martin Schwärzel, Berlin, Germany; and Amita Seghal, Chevy Chase, Maryland, US. *TyrR*^*f05682*^ (CG7431f05682, FBal0184987), *TyrRII*^Δ*29*^ (CG16766, FBgn0038541), and *TyrRII-TyrR*^Δ*124*^ were kindly provided prior to publication by Edward Blumenthal, Milwaukee, Wisconsin, US (Table 1). Receptor mutants and their respective control lines were outcrossed for at least 6 generations into Canton-S background.

**Table 1:** Fly strains used in this work.

Designation	Identifier	Associated target	Source or reference	Additional information
*t*β*h*^*nM18*^	FBal0061578	*t*β*h*	[42]	Gift from Henrike Scholz; not recently outcrossed
*t*β*h*^*nM18*^,UAS-*t*β*h*	FBti0038601		[69]	
UAS-*t*β*h*				Gift from Henrike Scholz
*hsp-t*β*h*	FBal0152162		[70]	Gift from Martin Schwärzel
*t*β*h*^*nM18*^;;*hsp-t*β*h*			[70]	Gift from Martin Schwärzel
*oamb*^*286*^	FBti0038368	oamb	[68]	Gift from Amita Seghal
*oamb*^*584*^	FBti0038361	oamb	[68]	Gift from Amita Seghal
*Oct*β*2R*^Δ*3*.*22*^	CG6989	*Oct*β*2R*	[71]	Gift from Martin Schwärzel before publication
*Oct*β*2R*^Δ*4*.*3*^	FBgn0038063	*Oct*β*2R*	[71]	Gift from Martin Schwärzel before publication
*honoka*	FBal0104701	TyrR	[72]	Gift from Andreas Thum
*TyrR*^*f05682*^	*CG7431*^*f05682*^, FBal0184987	TyrR	[73]	Gift from Edward Blumenthal
*TyrRII*^Δ*29*^	CG16766, FBgn0038541	TyrR	[73]	Gift from Edward Blumenthal
*TyrRII-TyrR*^Δ*124*^	FBab0048326	TyrR	[73]	Gift from Edward Blumenthal

The *t*β*h*^*nM18*^ mutation is thought to be a null allele and abolishes OA synthesis. Consequently, the precursor of OA, TA, accumulates to approximately 8-fold over control levels [42]. Mutants for *Oct*β*2R* were created using recombination of FRT-containing P-elements (Parks et al., 2004), as described elsewhere [71]. In order to obtain hetero- and hemizygote mutants, we crossed the *t*β*h*^*nM18*^ mutant line with its original control line, which was also obtained from Henrike Scholz.

### Fly care

Flies were kept on standard cornmeal/molasses food in a 12/12 h light/dark cycle at 60% relative humidity and 25°C except for *hsp*-*t*β*h* and elaV-GAL4;tub-GAL80 crosses, which were kept at 18°C without humidity control.

After hatching, experimental flies were collected into new food vials for 2 days. The day before testing, flies were CO_2_-anesthetized and sorted by sex (females except for UAS-*t*β*h* experiments), and their wings were clipped at two-thirds of their length. If not stated otherwise, animals recovered in the food vials overnight. Individuals were captured using a fly aspirator and transferred into the experimental setup on the following day.

### Heat-shock

*hsp*-*t*β*h* flies were heat shocked for 30–45 min at 37°C with 3–4 h of recovery time at 25°C before testing. elaV-GAL4;tub-GAL80;UAS-Tdc2 flies were heated at 33°C overnight, with 30 min of recovery time at room temperature before testing.

### Buridan’s paradigm

We used the Buridan’s setup to test fly locomotion; details are described in [57] (RRID:SCR_006331). Briefly, 2 black stripes (30 mm in width and 320 mm in height) were positioned opposite of each other 146.5 mm from the center of a platform (117 mm in diameter) surrounded by water and illuminated with bright white light from behind. The centroid position of the fly was recorded by custom tracking software (BuriTrack, http://buridan.sourceforge.net). If a fly jumped off the platform, it was returned by a brush, and the tracker was restarted. All data were obtained from 5 min of uninterrupted walk or the first 5 min of a 15-min walk. See doi: 10.17504/protocols.io.c7vzn5 for fly preparation.

Data were analyzed using CeTrAn v.4 (https://github.com/jcolomb/CeTrAn/releases/tag/v.4) as previously described in [57]. Briefly, walking speed was measured in traveled distance over time. A median was calculated for the progression of 1 experiment; the mean of all medians is reported in the graphs. Speeds exceeding 50 mm/s were considered to be jumps and were not included in the median speed calculation [57]. Stripe deviation acted as a metric for fixation behavior. It corresponds to the angle between the velocity vector and a vector pointing from the fly position towards the center of the frontal stripe (for details see [57]). Therefore, the larger the stripe deviation, the less accurately the fly fixated the stripe and vice versa. The platform inside the arena was cleaned with 70% ethanol after each experiment to minimize odor cues.

Buridan’s paradigm appears to be particularly sensitive to differences in genetic background [74]. Therefore, special emphasis was placed on always measuring all relevant genetic control lines simultaneously with the manipulated flies.

### Sugar sensitivity test

Sugar response was measured as described elsewhere [51]. Briefly, flies were starved for 20 h with Evian water. Flies were immobilized by cold anesthesia using a cold station (Fryka-Kälteteschnik, Esslingen am Neckar, Germany), and a triangle-shaped copper hook was glued to the head and thorax. Three hours later, the hook was attached to a rack so that free movement of flies’ tarsi and proboscis was enabled. A filter paper soaked with sucrose solution was presented to all the tarsi. The proboscis extension response to a serial dilution of sucrose (0%, 0.1%, 0.3%, 0.6%, 1%, 3%, and 30%) was recorded. The total number of the fly’s responses to all sucrose stimulations of increasing concentration was calculated [75]. Finally, the proboscis was stimulated by 30% sucrose solution. Flies not responding to proboscis stimulation or responding to the first stimulation (water only) were discarded from the analysis.

### Statistics

The mean walking speed was calculated out of medians (see “Buridan’s paradigm” above) and plotted with the standard error of the mean. Sucrose response and stripe deviation are shown as boxplots representing the median (bar), the 25%–75% quantiles (box), and data within (whiskers) and outside (outliers as black dots) 1.5 times interquartile range. Statistical analyses were performed in R (RRID:SCR_001905). Walking speed data followed normal distribution whereas stripe deviation did not (Shapiro–Wilk test of normality, *p* < 0.05), so we used the parametric 2-way ANOVA followed by Tukey HSD post hoc test and Welch 2-sample *t* test, respectively, or non-parametric paired Wilcoxon rank sum test with Bonferroni correction and Wilcoxon rank sum test, respectively. The *p*-value was additionally corrected for 2 repeated measurements of data from Buridan’s paradigm. The sample size of each group is indicated within the graphs. Default alpha value was set to 0.005 [76].

## Results

### Differential dominance of *t*β*h* mutation for different behavioral parameters

We examined the effects of the *t*β*h*^*nM18*^ mutation in 2 different experiments, assessing 3 different behavioral variables. We analyzed walking behavior in Buridan’s paradigm [57,58], reporting both the median speed (a temporal measure of behavior) and stripe deviation (as a spatial measure assessing object fixation). The second experiment quantified sugar responsiveness after 20 h of starvation using proboscis extensions [51].

**Fig 1:**
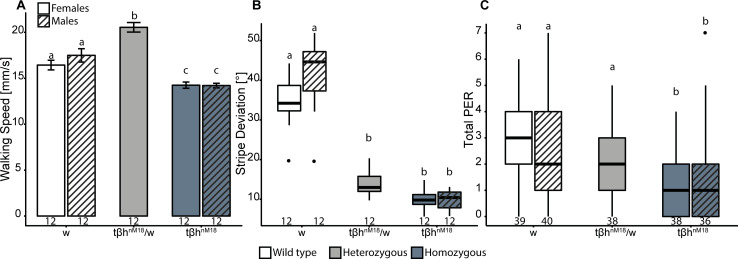
Differential dominance of the *t*β*h* mutation. Homo-, hetero-, and hemizygous *t*β*h* mutants and their controls were tested in Buridan’s paradigm and the sugar sensitivity test. (A) Median walking speed in Buridan’s paradigm. Homozygous *t*β*h*^*nM18*^ mutants walk more slowly than the wild-type controls, while heterozygous mutants walk faster than wild type (2-way ANOVA followed by Tukey HSD post hoc test, *p* < 0.005). Hemizygous mutant males walk more slowly than wild-type males (Welch 2-sample *t* test, *p* < 0.005). (B) Stripe deviation, a measure of stripe fixation during walking, is reduced in homozygous *t*β*h*^*nM18*^ mutants compared to heterozygous mutants and wild type (paired Wilcoxon rank sum test with Bonferroni correction for repeated measurement, *p* < 0.005) as well as in hemizygous males compared to their wild-type control (Wilcoxon rank sum test, *p* < 0.005). (C) The total number of proboscis extension responses (PER) to a serial dilution of sucrose after 20 h of starvation is depicted. Homozygous and hemizygous *t*β*h*^*nM18*^ mutants respond less to sucrose compared to wild-type controls; heterozygous mutants are not statistically different from wild type but different from homozygous mutants. Significant differences are tested by paired Wilcoxon rank sum test with Bonferroni correction (*p* < 0.005). In (A), bars and error bars indicate mean and standard error of the mean. In (B) and (C), the Tukey boxplots represent the median (bar), 25%–75% quartiles (box), and total data range (whiskers) excluding outliers outside of 1.5× interquartile range (dots). Numbers below graphs indicate sample size. Bars and boxes labeled with different letters are statistically significantly different. Raw data and evaluation code available at doi: 10.5281/zenodo.4568550.

Homozygous mutants behaved significantly differently from their genetic-background-matched control flies in all 3 measures: homozygous female mutants showed reduced walking speed (Fig 1A), fixated the stripes more closely (Fig 1B), and were less likely to extend their proboscis to a sugar solution after starvation (Fig 1C) compared to control flies with 2 intact *t*β*h* alleles. Flies heterozygous for the *t*β*h*^*nM18*^ mutation did not behave similarly homogeneously across the 3 observed variables. In walking speed, heterozygous flies with only 1 intact *t*β*h* allele exceeded wild-type animals by about 20% (Fig 1A), indicating overdominant inheritance. In stripe deviation, heterozygous flies behaved more similarly to the mutant flies than to the wild-type control flies, indicating dominant inheritance (Fig 1B). In the proboscis extension experiment, starved heterozygous mutant flies extended their proboscis a median 2 times, compared to a median 1 time for homozygous mutants and a median 3 times for the wild-type females (Fig 1C). Despite being halfway between the 2 homozygous groups, indicating intermediate inheritance, the heterozygote data are not statistically different from the wild-type controls, indicating a recessive phenotype.

As some aspects of Buridan’s paradigm have been shown to be highly sensitive to genetic background [74] and the stripe deviation for the w+ control strain appeared unusually large, we repeated the locomotion experiment using flies with a different genetic background. We examined *t*β*h* hemizygous mutants and control males resulting from a cross to another wild-type background. We found the same walking speed and stripe deviation phenotypes for *t*β*h* mutants in the w+/Canton-S background (*p* < 0.05, *n* = 34, Welch 2-sample *t* test for speed, Wilcoxon rank sum test for stripe deviation; data at doi: 10.6084/m9.figshare.1162439).

Taken together, we find that flies heterozygous for the *t*β*h*^*nM18*^ mutation provide evidence for 3 different modes of inheritance, depending on the phenotype analyzed, conforming to the definition of differential dominance.

### Driving *t*β*h* expression via GAL4-UAS

**Fig 2:**
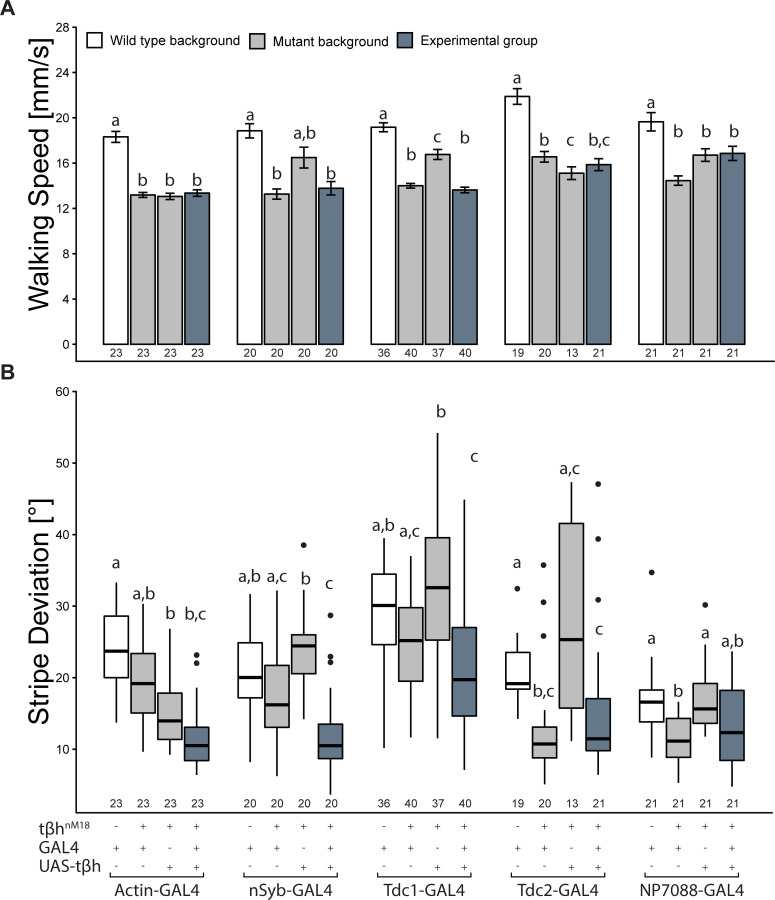
X-linked UAS-*t*β*h* expression cannot rescue the mutant Buridan phenotypes. (A) Median walking speed cannot be rescued by heterozygous GAL4-UAS-dependent *t*β*h* expression in mutants. All groups are different from the wild-type control, except for the UAS-*t*β*h* control in the nSyb experiment (2-way ANOVA with Tukey HSD post hoc test and correction for multiple measurements, *p* < 0.005). (B) Stripe deviation performance is already increased by the presence of the GAL4 or UAS construct. Ubiquitous Actin-GAL4 or pan-neuronal nSyb-GAL4 expression worsens the phenotype compared to the control lines (paired Wilcoxon rank sum test with Bonferroni correction, *p* < 0.005). In (A), bars and error bars indicate mean and standard error of the mean. In (B), the Tukey boxplots represent the median (bar), 25%–75% quartiles (box), and total data range (whiskers) excluding outliers outside of 1.5× interquartile range (dots). Numbers below graphs indicate sample size. Bars and boxes labeled with different letters are statistically significantly different. Raw data and evaluation code available at doi: 10.5281/zenodo.4568550.

What consequences can differential dominance have on standard genetic techniques commonly leveraged to understand gene function? To tackle this question, we started with a tried-and-tested method of transgenically expressing a wild-type version of the mutated gene in various tissues using the GAL4/UAS system [20–26,45]. As gene expression on the X chromosome is doubled in hemizygous males, which behave indistinguishably from female flies (Fig 1), the phenotypes of the heterozygous mutants (Fig 1) may be due to the reduced expression of the single intact *t*β*h* gene. In other words, the failure of the heterozygous flies to behave like wild-type flies may be due to the reduced gene dosage. To avoid such reduced gene expression in trans-heterozygous animals and to, instead, mimic the hemizygous wild-type males, we started our rescue experiments by driving an X-linked UAS-*t*β*h* transgene. We drove expression of the rescue construct in different tissues in *t*β*h* mutant males: in all cells (Actin-GAL4), in all neurons (nSyb-GAL4), in non-neuronal tyraminergic cells (Tdc1-GAL4), in tyraminergic neurons (Tdc2-GAL4), and in octopaminergic neurons (NP7088-GAL4). All of those lines drive expression throughout development and in adulthood (Fig 1). The X-linked transgene not only ensures doubled transcription from the single gene copy as in wild-type males, it is also more practical as it is situated on the same chromosome as the mutation that is to be rescued. In fact, this chromosome was engineered precisely to make such rescue experiments more convenient than with the rescue transgene on an autosome, which is not unusual in functional genetics. Because we have already successfully used this technique on sugar responsiveness [51], we focus on the walking measures from now on. Neither the temporal nor the spatial walking measure showed any rescue for any of the targeted tissues (Fig 2). In fact, for stripe deviation, some drivers yield even stronger stripe fixation than the mutant control strains (Fig 2B). In both measures, some of the lines carrying the rescue construct alone already fail to show the mutant phenotype. Superimposed on the general pattern of little effect on walking speed (Fig 2A) and a reduction of stripe deviation (Fig 2B), one can observe additional variability between the different groups. Presumably, this is due to the portions of differing genetic backgrounds the different GAL4 lines brought into the genotypes [74].

**Fig 3:**
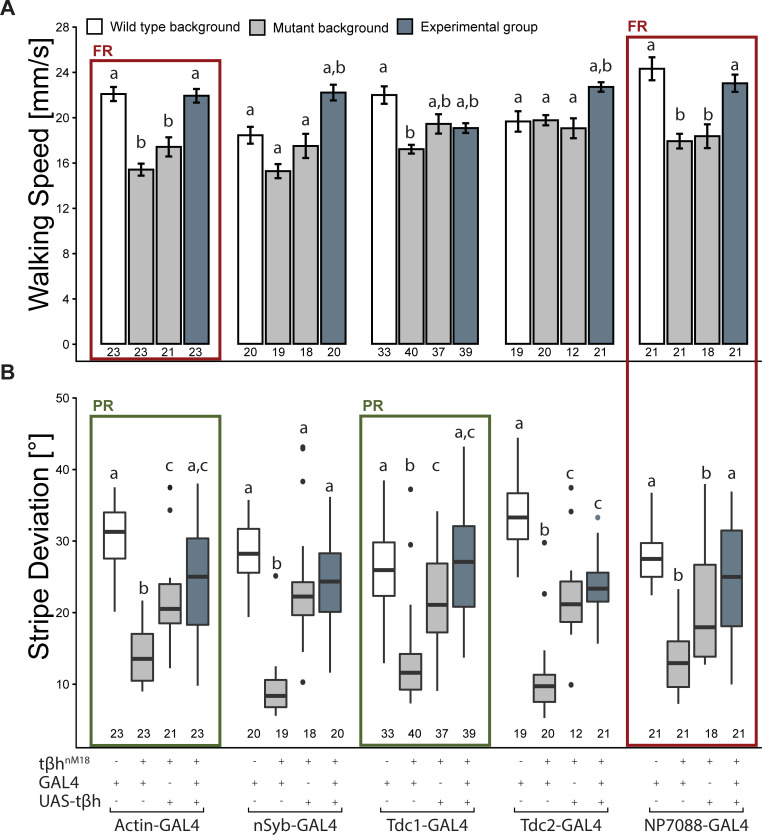
Autosomal UAS-*t*β*h* expression can rescue mutant Buridan phenotypes and phenocopy overdominance. (A) Median walking speed can sometimes be rescued by heterozygous GAL4-UAS-dependent *t*β*h* expression in mutants. Only expression in all cells (Actin-GAL4) and exclusively in octopaminergic cells via NP7088-GAL4 leads to a full rescue (FR, red boxes) of the walking phenotype, characterized by significant differences of the experimental line (blue) from both mutant control groups (grey), but not from the wild-type control (white) (2-way ANOVA with Tukey HSD post hoc test and correction for multiple measurements, *p* < 0.005). (B) Stripe deviation performance is already increased by the presence of the UAS construct. Only the octopaminergic NP7088-GAL4 rescues the stripe fixation phenotype. Expression of the transgene in all cells (Actin-GAL4) and in non-neuronal tyraminergic cells (Tdc1-GAL4) leads to a partial rescue (PR, green boxes), characterized by the experimental group failing to reach significant differences from either the wild-type control or one of the mutant controls (paired Wilcoxon rank sum test with Bonferroni correction, *p* < 0.005). In (A), bars and error bars indicate mean and standard error of the mean. In (B), the Tukey boxplots represent the median (bar), 25%–75% quartiles (box), and total data range (whiskers) excluding outliers outside of 1.5× interquartile range (dots). Numbers below graphs indicate sample size. Bars and boxes labeled with different letters are statistically significantly different. Raw data and evaluation code available at doi: 10.5281/zenodo.4568550.

We had speculated that the heterozygous results (Fig 1) may be due to low *t*β*h* transcription from the single gene dose. The rescue results (Fig 2), on the other hand, may indicate that expression of too much *t*β*h* may also disrupt the walking behavior. To test this hypothesis, we performed the exact same experiments again, but this time with the UAS-*t*β*h* transgene on the third chromosome (Fig 3), mimicking the situation in the heterozygous animals with halved gene expression, compared to the X-linked construct.

While again the mutant control strains with the rescue construct alone already showed some rescue effects, driving the rescue construct from the third chromosome yielded dramatically different results (Fig 3) compared to the X-linked rescue attempts (Fig 2). Octopaminergic (via NP7088-GAL4), but not tyraminergic (via Tdc-GAL4), expression led to a full rescue of both walking phenotypes, characterized by the rescue strain differing significantly from both mutant control strains but not from the wild-type control. Expression in all cells (via Actin-GAL4) rescued walking speed completely, but the stripe fixation phenotype was rescued only partially. The overdominance in walking speed observed in heterozygous mutants was phenocopied in the pan-neuronal driver (nSyb-GAL4) as well as in the neuronal tyraminergic driver (Tdc2-GAL4). In both lines, the presence of the GAL4 constructs appears to already lower walking speed compared to the other wild-type controls, making it indistinguishable from the mutant controls. Surprisingly, despite the expression pattern of Tdc2-GAL4 resembling that of NP7088-GAL4, there was no rescue of the stripe deviation phenotype, suggesting that stripe deviation is not influenced by different levels of TA in neurons. However, there was a partial rescue with the non-neuronal tyraminergic driver (Tdc1-GAL4), suggesting that non-neuronal tyraminergic cells (which do not express OA in wild-type animals) influence stripe fixation.

These results suggest that the differential dominance of the *t*β*h* gene seems to be related to gene dosage. However, any deviation from wild-type expression levels, whether a decrease or an increase, can lead to significant differences from wild-type behavior, rendering such standard experiments more of a lottery for pleiotropic genes that show differential dominance.

### Acute *t*β*h* expression differentially affects walking speed and stripe fixation

**Fig 4:**
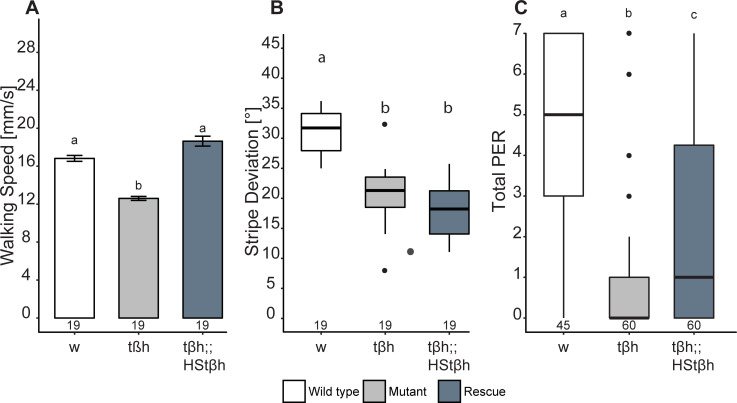
Acute and ubiquitous *t*β*h* expression rescues walking speed and sugar response but not stripe deviation. Temporal control of *t*β*h* expression was achieved by inducing transcription with a heat shock (HStβh) 3 h before the test. (A) Median walking speed is increased beyond wild-type levels after *t*β*h* induction (2-way ANOVA followed by Tukey HSD post hoc test, *p* < 0.005, *F* = 69.8). (B) Stripe deviation is not affected by *t*β*h* induction. (C) Sugar response (proboscis extension response [PER]) is increased by *t*β*h* expression, but does not reach wild-type levels (paired Wilcoxon rank sum test with Bonferroni correction, *p* < 0.005; data already published in Damrau et al. [51]). In (A), bars and error bars indicate mean and standard error of the mean. In (B) and (C), the Tukey boxplots represent the median (bar), 25%–75% quartiles (box), and total data range (whiskers) excluding outliers outside of 1.5× interquartile range (dots). Numbers below graphs indicate sample size. Bars and boxes labeled with different letters are statistically significantly different. Raw data and evaluation code available at doi: 10.5281/zenodo.4568550.

Another commonly used rescue technique is to ubiquitously express a wild-type variant of the gene in the mutant background after development in the adult fly, i.e., right before the experiment. In our case, we expressed the *t*β*h* gene in homozygous *t*β*h*^*nM18*^ mutant females under the control of the heat shock promoter *hsp*-*t*β*h*, situated on the third chromosome [69]. A heat shock was induced for 45 min at 37°C, and flies were allowed to recover for 3 h. After this treatment, rescue flies walked faster than controls (Fig 4A), phenocopying the overdominance results of the heterozygote flies (Fig 1A). These results did not quite reach our stringent 0.005 alpha threshold, but passed the 0.05 threshold for suggestive effects. Given the behavior of the heterozygous flies, it is straightforward to assume an analogous overdominance effect in this case. In contrast, expressing the *t*β*h* gene in this way left stripe fixation unaffected (Fig 4B), similar to how heterozygous flies’ stripe deviation was indistinguishable from that of homozygous mutant flies (Fig 1B). As published previously [51], sugar response after heat shock rescue was significantly improved (Fig 3C from [51]) without reaching wild-type performance, similar to how heterozygous flies show an intermediate number of proboscis extensions when compared with the 2 homozygous groups (Fig 1C).

Taken together, the results from heat-shock-induced expression of *t*β*h* in the mutant background (Fig 4) phenocopied those of the heterozygous flies (Fig 1) throughout. Possibly, using a *hsp*-*t*β*h* construct on the X chromosome may lead to a more successful rescue (i.e., opposite to the UAS rescue experiments). However, we are not aware of a *t*β*h*^*nM18*^,*hsp*-*t*β*h* X chromosome.

### Overexpressing *Tdc2* and *t*β*h* differentially affects walking speed and stripe fixation

**Fig 5:**
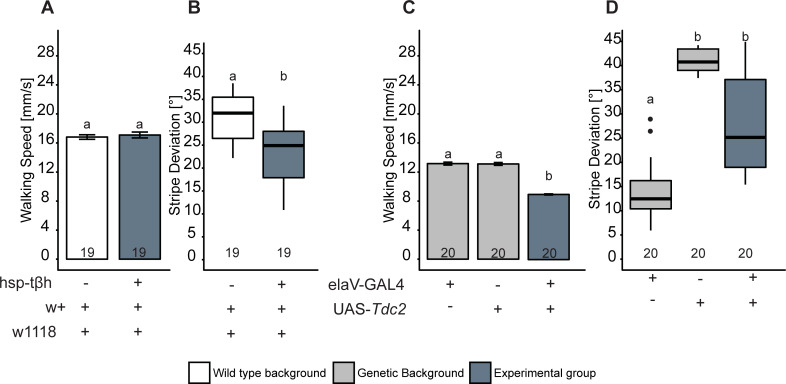
Acute overexpression of tyramine and octopamine synthesis enzymes in wild-type background affects behavior in Buridan’s paradigm. (A) Median walking speed is not affected when *t*β*h* is overexpressed in wild type 3 h before testing via *hsp-t*β*h* (Welch 2-sample *t* test, *p* < 0.005). (B) In contrast, stripe deviation is reduced when *t*β*h* is overexpressed (Wilcoxon rank sum test with correction for multiple measurements, *p* < 0.005). (C) Median walking speed is reduced after overexpression of *Tdc2* (2-way ANOVA followed by Tukey HSD post hoc test, *p* < 0.005). (D) The 2 parental controls show very different stripe deviation behavior, which prevents the interpretation of the performance of the overexpression group (paired Wilcoxon rank sum test with Bonferroni correction, *p* < 0.005). In (A) and (C), bars and error bars indicate mean and standard error of the mean. In (B) and (D), the Tukey boxplots represent the median (bar), 25%–75% quartiles (box), and total data range (whiskers) excluding outliers outside of 1.5× interquartile range (dots). Numbers below graphs indicate sample size. Bars and boxes labeled with different letters are statistically significantly different. Raw data and evaluation code available at doi: 10.5281/zenodo.4568550.

All experiments so far seem to suggest a very high sensitivity of the 3 chosen phenotypes to *t*β*h* gene dosage, where only a narrow range of gene expression supports wild-type behavior. To test this hypothesis, we increased the acute expression of the *t*β*h* and *Tdc* (tyrosine decarboxylase; synthesizes TA from tyrosine) enzymes in wild-type animals (Fig 5). While *t*β*h* overexpression is assumed to lead to OA production from TA and hence a decrease in TA titers, the *Tdc* overexpression should lead to increased TA production and hence a subsequent increase in OA concentration as well.

Overexpressing *t*β*h*, presumably decreasing TA levels and increasing OA levels, had no effect on walking speed (Fig 5A), but decreased stripe deviation (Fig 5B). *Tdc2* overexpression, presumably elevating both TA and OA above wild-type levels, reduced walking speed (Fig 5B), but yielded a stripe deviation phenotype in the middle of the (large) range of variation found in driver and effector lines.

These results hence suggest that overexpressing *t*β*h* selectively affected the spatial measure stripe deviation, while overexpressing *Tdc* seemed to mainly affect the temporal measure walking speed. These results support the hypothesis that the Buridan phenotypes are exquisitely sensitive to *t*β*h* gene dosage. They also raise the possibility that the mechanism by which this sensitivity is achieved involves the relative levels of TA and OA, mediated by *t*β*h* expression. In order to investigate this possibility in a way that is both *t*β*h* gene dosage independent and can separately manipulate TA and OA signaling, we tested a number of TA and OA receptor mutants.

### Differential involvement of OA and TA receptors in walking speed and stripe fixation

To specifically affect the signaling of only 1 of the amines independently of the *t*β*h* locus, we manipulated the OA/TA system on the receptor level and examined several OA and TA receptor mutants, all outcrossed to the same genetic background (see “Materials and methods”).

**Fig 6:**
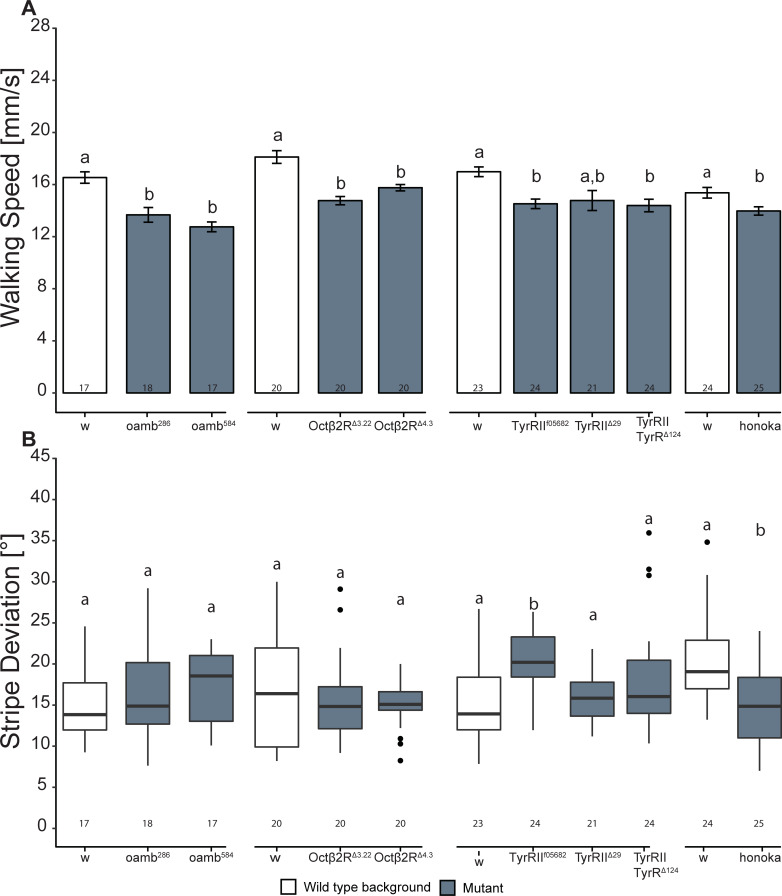
Differential roles of tyramine and octopamine signaling in Buridan’s paradigm. (A) *oamb*^*286*^, *oamb*^*584*^, *Oct*β*2R*^Δ*3*.*22*^, *Oct*β*2R*^Δ*4*.*3*^, *honoka*, and *TyrR*^*f05682*^ mutants and the double mutant *TyrRII-TyrR*^Δ*124*^ walk more slowly than their respective controls, while the walking speed of the *TyrRII*^Δ*29*^ mutant is indistinguishable from that of wild type (Welch 2-sample *t* test with correction for multiple measurements, *p* < 0.005). (B) Stripe deviation is not affected in octopamine receptor mutants. In *TyrR*^*f05682*^ and *honoka* mutants, stripe deviation is significantly increased. Significant differences between control and the respective receptor mutant are calculated by Wilcoxon rank sum test with correction for multiple measurements (*p* < 0.005). In (A), bars and error bars indicate mean and standard error of the mean. In (B), the Tukey boxplots represent the median (bar), 25%–75% quartiles (box), and total data range (whiskers) excluding outliers outside of 1.5× interquartile range (dots). Numbers below graphs indicate sample size. Bars and boxes labeled with different letters are statistically significantly different. Raw data and evaluation code available at doi: 10.5281/zenodo.4568550.

We tested 2 alleles for each of 2 OA receptors *oamb* and *Oct*β*2R*, as well as 1 allele each for the 3 TA receptors *honoka*, *TyrR*, and *TyrRII*, and a double receptor mutant for *TyrR* and *TyrRII*. While walking speed was affected in 7 mutants (Fig 6A; only the *TyrRII*^Δ*29*^ mutation had no effect), the stripe deviation was affected in only the 2 TA receptor mutants *TyrR*^*f05682*^ and *honoka*, in opposite directions (Fig 6B). Interestingly, the double mutant *TyrRII*-*TyrR*^Δ*124*^ showed no mutant phenotype in stripe fixation.

In principle, one would need to rescue each of the receptor mutants as well, to exclude effects from genetic variations outside of the targeted locus. With regard to the results of the rescue experiments shown here, we have abstained from such experiments and rely, instead, on the extensive outcrossing of the lines to homogenize the genetic background between mutants and controls, to decrease the chances of off-target differences.

The results of the receptor experiments suggest that OA is more specifically involved in walking speed, while TA signaling seems involved in both walking speed and stripe fixation. This latter hypothesis would be consistent with *Tdc* overexpression leading to reduced walking speed (Fig 5B) and Tdc1-GAL4-driven partial rescue of stripe fixation (in cells that do not express OA; Fig 3B), but only if these phenotypes are also sensitive to deviation from wild-type TA titers in both directions, increases and decreases.

## Discussion

### Differential dominance of the *t*β*h*^*nM18*^ mutation

A likely null mutation [42] for the X-linked synthesis enzyme of the biogenic amine OA, *t*β*h*^*nM18*^, showed differential dominance in 3 different behavioral traits (Fig 1). Pleiotropic alleles often show differential dominance [4,9], accompanied by overdominance in some of the traits [10,19]. However, it is not immediately obvious that the effects we observed are indeed attributable to differential dominance. Other phenomena that could lead to different outcomes for our different genotypes are dosage compensation in sex-linked genes (such as *t*β*h*) and other epigenetic effects, e.g., via differential maternal or paternal transfer of the gene in question (such as in our rescue experiments; Figs 2–4).

### Differential dominance is mediated by differential sensitivity to *t*β*h* gene expression levels

The *t*β*h* gene is located on the X chromosome (X:7,995,697..8,027,394). The homozygous females and hemizygous males show identical phenotypes in all parameters tested, both in the mutant and the wild-type genotypes (Fig 1). Thus, in these 3 cases, the presence of 1 or 2 X chromosomes appears to be irrelevant to the phenotype. Importantly, in males, the presence of 1 *t*β*h* allele is sufficient to provide the males with a wild-type phenotype for the parameters we studied. In the experiments where we test heterozygous females (i.e., with only 1 intact allele of *t*β*h*) and find phenotypes indicating differential dominance, no dosage compensation takes place as this process occurs in males in *Drosophila* and not in females [77–82]. Presumably, the levels of *t*β*h* gene expression may be lower in heterozygous animals than in either wild-type males or wild-type females. Importantly, this lower level of gene expression does not have the same effect in all phenotypes, leading to varying inheritance. Finally, the mutant allele in the heterozygous females always came from the mutant father, while a wild-type mother (w+, with matched genetic background) provided the other X chromosome, such that any heterogeneity in the inheritance cannot come from heterogeneity in parent-of-origin imprinting effects, either. Thus, the only remaining explanation from the design of these experiments is that the pleiotropic *t*β*h* locus indeed confers differential dominance to the alleles we used here (Fig 1), likely mediated by differential sensitivity of the 3 behaviors to *t*β*h* gene expression.

### Differential sensitivity to *t*β*h* gene expression levels is mediated by a TA/OA opponent system

Several studies have suggested that OA and TA may operate as an opponent system [47,83–86]. If this is the case, the raised levels of TA with the *t*β*h*^*nM18*^ allele, rather than the lower OA levels, may be partly responsible for some of the phenotypes observed. To our knowledge, it is still unknown if *t*β*h* is indeed the rate-limiting enzyme for OA synthesis or what effects manipulations of *t*β*h* expression levels may have on actual OA/TA titers. However, given that the OA precursor TA is also involved in locomotor control, it is straightforward to speculate that the acute sensitivity to *t*β*h* gene expression levels we have observed here may be reflecting a sensitivity to actual OA/TA titers in the neuronal networks involved. Thus, one potential mechanistic explanation for the differential dominance of the *t*β*h* locus is that the neuronal networks controlling the non-recessive behavioral parameters are modulated by a TA/OA opponent system that confers a high sensitivity to the relative amine titers (and hence gene expression levels) to network function.

Both the spatial rescue (Figs 2 and 3) and the overexpression results (Fig 5) appear to support this hypothesis: We observed a partial rescue of stripe fixation in non-neuronal tyraminergic cells that do normally not release OA (Fig 3B), and overexpressing the TA-synthesizing enzyme *Tdc* affected walking speed.

To further explore this possibility, we manipulated TA and OA signaling individually, via OA receptor knock-out in the non-recessive walking parameter experiments (Fig 6). In these experiments, stripe fixation measures a spatial property of walking behavior, as the flies orient themselves towards the stripes in space. Walking speed, in contrast, is taken as one of several measures of the temporal control of walking behavior in Buridan’s paradigm. These 2 parameters commonly separate not only in principal components analyses, but also in biological manipulations.

#### Stripe fixation

All our X-linked GAL4/UAS manipulations of the *t*β*h* gene increased fixation behavior of the flies, even beyond wild-type levels. Perhaps most strikingly, median stripe deviation was the lowest value for every single driver line we tested (Fig 2B). This effect was also observed when driving gene expression with a heat shock construct from the third chromosome (Fig 4B). Only presumably lowering the levels of gene expression using a UAS construct on the third chromosome provided some successful rescue results. As part of such fixation behavior can be interpreted as an outcome of a fly’s light/dark preference [87], this dependence on gene expression levels may be understood by looking at photopreference results. Gorostiza et al. [87] discovered a correlation between dark preference in a T-maze and tighter fixation behavior in Buridan’s paradigm. With inhibited octopaminergic neurons, transgenic flies showed a lower dark preference, while activated octopaminergic neurons increased dark preference. It is conceivable that both the doubled *t*β*h* gene expression from the dosage-compensated X chromosomes (Fig 2B) and the *hsp*-driven rescue (Fig 4B) increased the dark preference in these flies analogously to the activation of tyraminergic/octopaminergic neurons in [87].

Indeed, in our array of receptor mutants tested, OA receptor mutant flies do not fixate the stripes any different from control flies, while flies mutant for the TA receptor *TyrR*^*f05682*^ fixate the stripes less strongly than wild-type controls (Fig 6B). In other words, decreased TA signaling can lead to decreased stripe fixation (Fig 6B), while the increased TA levels in *t*β*h* mutants [42] can explain some of the increased fixation behavior in these flies (Fig 1B). Although we have not tested all known OA receptors [88], considering the TA receptor and other data on stripe deviation, this suggests that TA may act independently of OA on this behavioral trait, with an increased TA activity leading to stronger stripe fixation. We thus conclude that our manipulations of the OA synthesis enzyme *t*β*h* affected stripe fixation, at least in part, via an involvement of the OA precursor TA. This conclusion suggests that in *t*β*h*^*nM18*^ mutants, the increased stripe fixation may be due to the elevated levels of TA, while in our rescue and overexpression experiments, it may be due to high levels of OA, corroborating the hypothesis that TA/OA opponent organization may be the mechanism underlying the observed differential dominance effects.

#### Walking speed

The contrast to stripe fixation (a spatial measure of walking behavior) could not be starker in walking speed (a temporal measure of walking behavior). While it proved exceedingly difficult to decrease stripe fixation to control levels (observed in only 1 out of 13 manipulations), adding or removing *t*β*h* genes both increased and decreased walking speed. For instance, removing 1 copy (i.e., in the heterozygous state) increased walking speed, while removing both (homozygous mutants) decreased walking speed (Fig 1A). Confirming the general observation that these 2 behavioral parameters are separable, also in these experiments walking speed and stripe fixation are decoupled.

Some lines driving transgenic expression of autosomal *t*β*h* rescue constructs (Fig 2A), as well as acute *t*β*h* rescue before the experiment (Fig 4A), yielded a phenocopy of the heterozygous flies: walking speed increased beyond that of wild-type controls. At the same time, all X-linked rescue experiments failed to increase walking speed beyond mutant levels, suggesting that lower than normal *t*β*h* expression increases walking speed, and higher than normal levels decrease it. This hypothesis is supported by our overexpression results: *Tdc2* overexpression throughout development reduces walking speed (Fig 4A). The overexpression results in walking speed are mirror-symmetric with those in stripe fixation, supporting the potential opponent role OA and TA may be playing in both parameters and hence their role in establishing differential dominance in the *t*β*h* locus.

Due to these opposite results between spatial and temporal control of walking behavior and the highly varying nature of the walking speed results, one may speculate whether walking speed is controlled by OA alone or by OA in conjunction with TA. As we find that both OA and TA receptor mutants are affected in walking speed (Fig 6A), we conclude that both OA and TA signaling are involved in the control of walking speed.

### Gene dosage in opponent systems

Manipulating *t*β*h* expression modifies the balance of OA and TA in opposite directions [42]. Therefore, the acute *t*β*h* gene expression dependence manifesting itself in differential dominance may be explained by the alteration of a fine balance between relative TA and OA concentrations. In *Drosophila* larvae, it was suggested that the relative increase in OA levels, but not the absolute endogenous amount, is important for regulation of starvation-induced locomotion [86]. However, the interaction between the 2 neuromodulators seems to be more complex than a simple balance [47,48,70,83–85,89–91]. We thus find that the data presented here are consistent with the hypothesis that one potential mechanism behind differential dominance in some traits is an opponent system of gene products that confers a high sensitivity to gene expression levels to these traits. These results support the hypothesis that the *t*β*h* gene exhibits type II pleiotropy [8].

### Differential dominance affects the outcomes of standard genetic techniques

As we have shown, this high sensitivity poses some formidable challenges for standard functional genetics techniques. A staple in the genetic toolbox is rescue experiments, which serve to establish the spatiotemporal expression requirements of the gene in question for the phenotypes under scrutiny (e.g., [23,24,45]). Such experiments are commonly carried out in order to arrive at necessity and sufficiency statements from which further mechanical understanding of gene function can follow (but see also [92,93]). However, the implicit and all too often untested assumptions for these experiments are that the (commonly) null mutations to be rescued follow recessive inheritance and that wild-type-level gene function can be restored with a single wild-type allele. The GAL4/UAS system does not provide for sufficient control of gene expression levels to accommodate more unconventional modes of inheritance. In fact, in some cases, the basal promoter used in the creation of the GAL4 line may decide about the success or failure of an experiment [94].

In this work, we not only introduced the wild-type allele of the *t*β*h* gene in its genomic locus in heterozygous animals (and hence with certainly wild-type spatiotemporal expression levels; Fig 1), but we also deployed commonly used spatial (Figs 2 and 3) and temporal (Fig 4) transgenic rescue techniques, as well as transgenic overexpression in a wild-type background (Fig 5). While failed rescue experiments typically indicate that the mutated gene is not involved in the observed phenotype, the aggregate of all our experiments suggests that indeed the *t*β*h* gene is involved in all the phenotypes we studied, despite multiple failed rescue experiments in the walking phenotypes. Specifically, the autosomal or gonosomal location of the rescue construct affected rescue results via male dosage compensation (Figs 2 and 3), but the choice of technique driving the rescue construct, inasmuch as it affects expression levels, was also important irrespective of its autosomal location (Fig 4). These data suggest that differential dominance can affect the outcome of some of these standard experiments to such an extent that nearly any arbitrary result may be obtained simply by the choice of rescue strategy—and the differential reporting of such results (file drawer effect) may distort the literature.

While pleiotropy was not found to be universal [8], it is not known how many genes in *Drosophila* are pleiotropic, nor how many of them display differential dominance. However, we have recently observed differential dominance in at least 1 other gene, the transcription factor *FoxP* [94].

## Abbreviations

OA: octopamine
TA: tyramine
